# Pseudobladder Sign: A Sign but Not Pseudosign

**DOI:** 10.31662/jmaj.2023-0128

**Published:** 2023-11-16

**Authors:** Steven H. Yale, Halil Tekiner, Eileen S. Yale

**Affiliations:** 1 University of Central Florida College of Medicine, Orlando, United States of America; 2 Department of the History of Medicine and Ethics, Erciyes University School of Medicine, Melikgazi, Turkey; 3 Division of General Internal Medicine, University of Florida, Gainesville, United States of America

**Keywords:** Sign, Pseudobladder, Double-bladder, Fothergill, Rectus Sheath Hematoma

## To the Editor:

We read with interest the case by Shiba et al. on “Giant rectus sheath hematoma: pseudobladder sign” and seek to address several aspects of this case ^(1)^. Coughing caused by coronavirus 2019 (COVID-19) was the proximate cause of this patient’s rectus sheath hematoma (RSH). Coughing, anticoagulation therapy, recent surgery, pregnancy, and medication injections, not COVID-19, are risk factors for RSH ^(2)^. The patient had abdominal wall tenderness and rebound tenderness similar to the peritoneal signs. However, referring to them as peritoneal signs would be incorrect because the hematoma was confined to the abdominal wall, not the peritoneum.

William Edward Fothergill (1865-1926) described a method for recognizing swelling caused by a hematoma involving the lower portion of the rectus sheath in the abdominal wall ^(3)^. He found that the swelling was palpable and fixed when the rectus muscle contracted and that there was resonance on deep percussion when the abdominal wall mass was not excessively large. Fothergill reported that RSH is found in postmenopausal women and occurs secondary to trauma and in those with coughing or vomiting ^(3)^. He recognized the limitation of this sign as other conditions may mimic a RSH, including an intrabdominal mass adherent to the abdominal wall and urachal cyst ^(3)^.

“Pseudo” is the Greek term meaning false. It would be incorrect to refer to this finding as a “pseudobladder” because, on ultrasound, the hematoma from the RSH is hyperechoic and clearly distinguishable from the adjacent anechoic urine in the bladder. It is a “sign” as it makes an inference about the underlying disease and provides a method that assists in diagnosis. In contrast, the “double-bladder sign” indicates that the urinary bladder and ovarian cysts are anechoic on ultrasound ^(4), (5)^. Therefore, it would be accurate, although the author did not call it such, to refer to this as a “pseudo double-bladder” as both structures appear similar on ultrasound. They differ because the ovarian cyst is not part of the bladder, although it appears as such. Additionally, it lacks the features of a sign, as determined radiographically, because the two structures are indistinguishable.

## Article Information

### Conflicts of Interest

None

### Author Contributions

Steven H. Yale, Halil Tekiner, and Eileen S. Yale substantially contributed to the concept of the work, drafted the work or critically reviewed it for important intellectual content, approved the version for publication, and agreed to be accountable for all aspects of the work to ensure that questions related to the accuracy or integrity of any part of the work were appropriately investigated and resolved.
